# Future contingency and God’s knowledge of particulars in Avicenna

**DOI:** 10.1080/09608788.2022.2088469

**Published:** 2022-06-28

**Authors:** Jari Kaukua

**Affiliations:** Department of Social Sciences and Philosophy, University of Jyväskylä, Jyväskylä, Finland

**Keywords:** Avicenna, future contingency, determinism, God's knowledge

## Abstract

Avicenna’s discussion of future contingent propositions is sometimes considered to entail metaphysical indeterminism. In this paper, I argue that his logical analysis of future contingent statements is best understood in terms of the epistemic modality of those statements, which has no consequences for modal metaphysics. This interpretation is corroborated by hitherto neglected material concerning the question of God’s knowledge of particulars. In the *Taʿlīqāt*, Avicenna argues that God knows particulars by knowing their complete causes, and when contrasted with the human knowledge of particulars, this epistemically superior access shows that the contingency of statements about future particulars is not due to the modal properties of real particulars but to the nature of human access to them.

It is a matter of ongoing debate whether Avicenna (d. 1037 CE) endorsed necessitarianism – the view that all that is, is necessarily so and that nothing whatsoever could be otherwise than it is. A strong piece of evidence for an affirmative answer is the fact that he articulates and explicitly commits to what modern philosophers call the principle of sufficient reason, when he holds that all that exists does so by virtue of a complete cause (*ʿilla tāmma*) that necessitates its existence.^1^ On the other hand, Avicenna’s necessitarianism has been contested by referring to his realism about contingency, which is especially evident in his characterization of all entities apart from God as merely possible by virtue of their essences, and necessary only by virtue of extrinsic causes (Goodman, *Avicenna*, 81). Scholars have also argued that there is room for indeterminacy in Avicenna’s theory of efficient and material causation (Rashed, “Théodicée et approximation”, and Ivry, “Destiny Revisited”),^2^ and that determinism is excluded by his commitment to voluntarism in ethics and theory of action (Janssens, “The Problem of Human Freedom”; and Ruffus and McGinnis, “Willful Understanding”).^3^

In addition to the foregoing, it has been suggested that Avicenna’s views on future contingents and God’s knowledge of particulars are incompatible with necessitarianism. In a seminal paper on Avicenna’s modal logic and metaphysics, Allan Bäck argues that Avicenna rejected Aristotle’s temporal (or statistical) interpretation of modality, which identifies necessity with actualization always, possibility with actualization at least once, and impossibility with actualization never, and allowed instead for both singular and universal possibilities that are never actualized in extramental existence. Consequently, Avicenna also rejects the inference from the truth of statements about future contingent things to logical determinism. On these grounds, Avicenna is not a logical determinist, even if he is a causal determinist, which Bäck takes to mean that he is not a necessitarianist (Bäck, “Avicenna's Conception of the Modalities”).^4^ On similar lines, in a paper on Abū Naṣr al-Fārābī’s (d. 950/1) treatment of the problem of future contingents, Peter Adamson mentions in passing that following al-Fārābī, Avicenna denies the ancient claim that the principle of bivalence, when applied to future contingent statements, entails necessitarianism. Like Bäck, Adamson argues that this is because Avicenna endorses a non-statistical theory of modality that recognizes unrealized possibilities, which is sufficient to rule out necessitarianism (Adamson, “The Arabic Sea-Battle”).

In the same paper, Adamson shows that al-Fārābī connected the discussion concerning future contingents to the question of God’s foreknowledge of particulars. This connection is of obvious relevance, for if God knows particulars prior to their coming to exist, it seems that they cannot fail to exist, and the question of whether the truth of future contingent statements entails necessitarianism becomes redundant. For this reason, al-Fārābī came up with the idea that created things are contingent in themselves, and that consequently, statements about future contingent things are contingently true or untrue, even though God foreknows the contingent things. (Adamson, “The Arabic Sea-Battle”, 180-184.)

According to the prominent view first presented by Michael Marmura, Avicenna solved this problem in a different way – if indeed he even saw it as a problem. Marmura’s seminal study took its cue from Avicenna’s formulation in the *Ilāhīyat*, according to which God knows particulars but “in a universal way” (*ʿalā naḥwin kullī*; Avicenna, *Ilāhīyāt* VIII.6.15, 288). According to Marmura, this means that God only knows the *kinds* of thing there are, not the numerically distinct individual instantiations of those kinds. God does know some individual things by accident, however, namely the celestial souls, bodies, and intellects, because they are the sole instantiations of their species. Consequently, He also knows some individual events, for the celestial phenomena are necessary concomitants of the celestial souls and bodies that He knows by accident. Importantly, however, God does not know sublunary individual substances or events, and consequently, His knowledge does not violate the indeterminacy and radical contingency of the sublunary part of the world. (Marmura, “Some Aspects of Avicenna’s Theory”; and Adamson, “On Knowledge of Particulars”.) Obviously, this theory of God’s knowledge of particulars does not exclude necessitarianism, but when considered in the light of al-Fārābī’s earlier connection between foreknowledge and necessity, it seems ideally suited to avoiding it.

In this paper, my objective is to argue that neither Avicenna’s solution to the ancient problem of future contingents nor his theory of God’s knowledge of particulars poses a problem for a necessitarian interpretation of his metaphysics. My claim is that when Avicenna denies that the truth of future contingent statements entails the necessity of the states of affairs to which they refer, he is not making a point about metaphysical modality, but about epistemic modality, understood as a property of statements. In other words, he is only saying that the meanings of the terms constituting the future contingent statement are not sufficient to determine the truth of the statement, and that instead, the statement’s truth can only be determined by means of its correspondence to the factual state of affairs it signifies – which has not been realized yet. The question of metaphysical necessity, or the question of necessitarianism, is left entirely open.

Regarding the question of God’s knowledge, I will supplement the seminal texts from the *Ilāhīyāt* with parallel material from the *Taʿlīqāt*, where Avicenna says that God not only knows particular things in a universal way but also by knowing their causes. Once this initially Fārābīan idea is spelled out, it becomes clear that Avicenna is fully aware of its necessitarian consequences, and most importantly, does nothing to mitigate them. Moreover, contrasting God’s way of knowing future individual things with our way of knowing them provides further evidence for the view that the contingency of those things is exclusively epistemic.

In the following, my focus will be strictly on these two questions, and for this reason I will not present an independent positive argument for necessitarianism here. Instead, I refer the interested reader to the scholarship cited above, the conclusion of which I believe stands once the alleged challenge of future contingents has been met.

## The truth of future contingent statements

Avicenna discusses future contingent statements at greatest length in *ʿIbāra* I.10, a chapter devoted to logical contradiction in different types of sentence. He begins by characterizing contradiction as

a difference between two propositions in terms of affirmation and negation such that it follows from the difference, by virtue of its essence, that one of them is true and the other false, either on its own (*bi-ʿaynihi*) or not on its own.

(Avicenna, *ʿIbāra* I.10, 67.)

This characterization posits two conditions for a contradictory pair of statements: (1) one of them must be an affirmation and the other a negation, and (2) the logical relation between them must be such that one and only one of them is true, that is, that the principles of bivalence and the excluded middle hold of the pair of contradictories. Avicenna also introduces a qualification concerning the reason why one of the contradictories is true (and the other false): it either is or is not such “on its own”. I take this to mean that a contradiction holds between a statement and its negation regardless of whether the statement is true or false considered in isolation, or simply by virtue of the intensional meanings of its terms;^5^ in other words, the disjunction (*P* ˅ ∼*P*) would be logically true, even if it were not determined whether *P* or ∼*P* is true.

Now, statements about future contingents are statements about individual things. This means that criterion (2) is relatively straightforward: a pair of contradictory future contingent statements is formed by simply qualifying the same predicative formula by affirmation and negation, as for instance in, “Zayd will walk tomorrow” and “Zayd will not walk tomorrow” (Avicenna, *ʿIbāra* I.10, 67).^6^ This is for two reasons. First, the subject term of a future contingent statement denotes a determined individual.^7^ This is obvious in the case of a proper name like ‘Zayd’, but it would also be true of a deictically qualified description, such as ‘this person’ or ‘this piece of wood’. Although this may seem a trivial observation, we will see that the deictic reference to an individual is a necessary condition of future contingency.

Another reason for the relative straightforwardness of contradiction in the case of future contingent statements is the reference they include to a specific time, like tomorrow in our two examples. This reference is crucial, for if it is removed, the two statements will no longer be contradictory: it can be simultaneously true that Zayd will walk and that he will not walk, for Zayd may well walk and stand still at different times in the future (Avicenna, *ʿIbāra* I.10, 67-68).

After these preliminary remarks, Avicenna turns to discuss how the truth values are determined in different kinds of contradictory pairs. In the case of quantified statements, “truth and falsity are determined (*yataʿayyanu*) by the proposition itself and by the nature of the matter” (Avicenna, *ʿIbāra* I.10, 70). To put this another way, some quantified statements (such as ‘all human beings are rational’) are true intrinsically (‘by the proposition itself’), or by virtue of the intensional meanings of the terms constituting the statement, because the predicated attribute is entailed by the essence denoted by the subject term. By contrast, other quantified statements are true on extrinsic grounds, namely, by virtue of their correspondence to factual states of affairs (or ‘by the nature of the matter’), such as ‘some human beings can finish the marathon in two hours’.^8^

Like quantified statements, individual statements about past and present states of affairs are also determined to be either true or false: “The same holds of tensed (*al-zamānīya*) individual propositions, the tenses of which are past or present, for a time that has taken place makes one thing’s conjunction to the nature of the other necessary.” (Avicenna, *ʿIbāra* I.10, 70.) However, there is a difference between the quantified and the individual statements, for whereas quantified statements may be true (or false) by virtue of the essences signified by the terms, Avicenna only says that individual statements are true (or false) by virtue of whether they correspond to factual states of affairs.^9^ In the case of individual statements in past and present tense, these factual states of affairs have already taken place and are thus capable of determining the truth value of the statement. It is possible, at least in principle, to appeal to them in determining whether the attribute signified by the predicate term is appropriately related to the individual signified by the subject term.

Now, the core question for us is whether the principle of bivalence holds similarly in the case of statements concerning *future* states of individual things. Avicenna writes:

When it comes to contradictory individual propositions concerning future affairs, in their case it is [i] *not necessary with respect to the natures of the affairs* that either truth or falsity be determined (*yataʿayyanu*) for them, nor has either of them been determined by virtue of [ii] the *occurrence of the determining cause* (*al-sabab al-muʿayyin*). [iii] The determination is either by virtue of what the affair necessitates in itself or, in the case of what is not necessarily determined by its essence, by virtue of the existence of the determining cause. Every necessary thing is either necessary by virtue of its essence or necessary by virtue of the occurrence of the cause which necessitates it.

(Avicenna, *ʿIbāra* I.10, 70-71; my emphases.)

Three points are worth noting in this loaded passage. First ([i]), Avicenna says that unlike its past and present counterparts, a future state of affairs cannot determine the truth of a statement about an individual in the future, and this is for the simple reason that the state of affairs does not obtain yet. This means, however, that the state of affairs signified by a future contingent statement is not necessary by virtue of itself, and consequently, the truth value of the statement signifying it cannot be determined simply by considering the conceived state of affairs in isolation from its realization. To use our previous example, as a healthy human being, Zayd will have the capacity to walk tomorrow, but the capacity alone does not determine whether Zayd will take a walk tomorrow. In this sense, the attribute signified by the predicate is accidental to the subject, and its occurrence is genuinely contingent, when considered with respect to the subject in isolation.

This brings us to our second point ([ii]). The state of affairs signified by the future contingent statement requires a determining cause for its occurrence or its failure to occur, and unless the determining cause obtains, or the necessary conditions for its obtaining tomorrow now obtain, it is not yet settled whether the state of affairs will obtain. It is not clear from Avicenna’s dense formulation whether the absence of the determining cause is a matter of our epistemic limitations or of genuine metaphysical indeterminacy, but I believe there are grounds to think that he is speaking of truth-determination, and thus of an epistemological concern, instead of the metaphysical question of necessitation. If prior circumstantial conditions, together with unchanging metaphysical principles, constitute the complete necessitating cause of every state of affairs, then it is easy to see that the presently obtaining states of affairs necessitate even a more distant future state of affairs, albeit through a number of intermediate states of affairs, one of which includes the proximate determining cause of that future state of affairs. To put this another way, causes earlier in the causal ancestry that leads to the future state of affairs do already obtain, and they are sufficient to necessitate the determining proximate cause of that state of affairs, and thereby the state of affairs itself. None of this information is included in the future contingent statement, however, and this for the good reason that such causal chains tend to be very complicated and thus an ill fit for concise statements. Since no mention of the relevant causal ancestry is made in the statement about the future state of affairs, the terms of the proposition yield no information about the occurrence of the proximate cause. Thus, looking at the statement in isolation, there is no way to determine whether the future contingent statement is true or false prior to its occurrence.^10^

Finally ([iii]), Avicenna recapitulates that the only two ways for determining the truth value of a factual statement are “by virtue of what the affair necessitates in itself” and by virtue of an extrinsic determining cause. In other words, if the statement is not true by virtue of a relation of constitution or implication between the intensional meanings of the subject and the predicate term, it must be true by virtue of a determining cause that is extrinsic to the state of affairs constituted by those two meanings. Now, a contingent statement is such precisely because it does not mention the cause that determines whether the predicate holds of the subject. To put this another way, the truth value of a contingent statement is always determined by a cause extrinsic to the meaning of the subject term, and never simply by the relation between its terms – otherwise it would no longer be a contingent statement.

## Future contingency and necessitarianism

This is the basis on which Avicenna raises the question we are interested in: does the principle of bivalence, conceived as applicable to *all* pairs of contradictories, entail necessitarianism when applied to pairs of contradictory future contingent statements? On one crucial supposition, this would be the case:

If the propositions, which we are discussing, were determined [to be] true or false, such that every affirmation and negation were either true or false *on its own* (*bi-ʿaynihi*), then every affair in the future would either inevitably exist or not exist.

(Avicenna, *ʿIbāra* I.10, 71; my emphasis.)

If all statements had their truth-value intrinsically and considered in isolation (‘on their own’), or in other words, if all statements were true or false simply by virtue of the relations of constitution or concomitance between the essences denoted by their terms, then it would be the case that all states of affairs denoted by these statements would either obtain necessarily or fail to obtain necessarily.

For Avicenna, this inference is invalid, because it builds on the supposition that the truth or falsity of future contingent statements can be determined before the denoted states of affairs have taken place (Avicenna, *ʿIbāra* I.10, 71). As explained in the foregoing, this would mean that a future contingent statement is true or false simply by virtue of the intensional meanings of its terms, or independently of the cause that necessitates the state of affairs denoted by the statement. This is misguided, because for genuinely contingent statements about individuals, the direction of fit can only be from the world to the statement:

The affair does not come to exist because [the statement] was true of it. Instead, the statement is true because the affair is such in itself. The necessity would take place in actual matter of fact, even if nothing were said.

(Avicenna, *ʿIbāra* I.10, 72.)

Our factual statements about the world are true because of the way the world is, and not the other way round. This means that we must give up the assumption that the truth of future contingent statements is determinable apart from the extrinsic cause on the grounds of which the predicate holds of the subject. Consequently, although the disjunction of the two future contingent contradictories is necessarily true, each disjunct considered in isolation is only possibly true (Avicenna, *ʿIbāra* I.10, 72-73). In other words, the principles of bivalence and excluded middle do hold of contradictory *pairs* of future contingent statements, but until the causes determining their respective truth values are introduced, the distribution of truth values will remain undetermined. The truth of the disjunction of such a contradictory pair only allows the conditional inference that if a future contingent statement *P* is true, then ∼*P* is false, and the other way round.

Nowhere in the foregoing does Avicenna signal that he is making this point to save indeterminism from the threat that results from applying the principle of bivalence to future contingent statements. Instead, he has just characterized the state of affairs denoted by the relevant statement as necessary independent of what is said about it. Later, however, Avicenna introduces apparently compelling evidence against the necessitarianism, which would follow from the determined truth value of future contingent statements. He begins by pointing to our certainty of being able to affect future courses of events by our own acts:

[W]e know that there are affairs that come to be by coincidence as well as things, which take place and do not take place in equal measure. Were that not the case, we would not have to deliberate, think, or prepare, believing that if we do what is necessary, something will take place that would not take place, were we to refrain. If the thing, about which we deliberate and for which we prepare, took place necessarily or did not take place necessarily, such that if someone said something about it and [this] was either true or false, the judgment [about the matter] being determined for his statement (*fa-yuʿayyanu ḥukmuhu li-qawlihi*), our preparation and deliberation would not be useful at all. Our intellects, however, testify to the usefulness of preparation, and we have no doubt about it. Hence, what refutes or falsifies it is absurd. Since some affairs are of this sort and there is preparation and concern over states different from them, they are not necessary by themselves (*ḍarūrīyan bi-nafsihi*) and not determined. Hence, this determination regarding truth and falsity is absurd.

(Avicenna, *ʿIbāra* I.10, 73.)

Following Aristotle’s lead (*De int.* 9, 18b25-19a15), Avicenna points to our intuitive certainty of the fact that our voluntary acts, based on careful deliberation about the various possible courses of action and the relevant circumstantial factors, can make a difference. Intuitive certainty of this sort carries greater weight than the suspicious inference of determinism from the truth of future contingents, and so the latter must go. Interestingly, Avicenna also thinks that parallel evidence can be drawn from natural events that allow for variation:

This does not only hold of affairs that take place by choice, but also of things that are by nature, like wood, for it is possible in its nature to remain until it decays, but it is also possible that fire meets and burns it, and neither of the affairs is necessary for it, insofar as it is wood.

(Avicenna, *ʿIbāra* I.10, 73.)

The fate of a piece of wood is not determined by its nature, for its being wood allows it to be burnt or to slowly rot away. And yet, if a statement like ‘this piece of wood will be destroyed by burning’^11^ were true *now*, this scope of evident possibility would be removed. Hence, statements of this sort cannot have a determined truth value.

At this point, it may seem that Avicenna rejects the application of the principle of bivalence to future contingent statements for the very reason that it leads to determinism, which is contradicted by the indubitable freedom of our will and the fact that the natures of things do not exhaustively determine their future fates. If this were true, his discussion of future contingents would constitute a strong case against those scholars, including myself, who hold that he endorses necessitarianism. On the other hand, in other places Avicenna clearly states that both our voluntary acts and natural events are necessitated by their causes.^12^ As regards voluntary agency, he does hold that it is free in the sense that it is not determined through extrinsic causes. This does not mean, however, that it is not determined by anything, for our capacity of volition can only initiate an act if it is determined to do so by a cognition concerning some possible end of action, that is, by a motive. In this sense, voluntary agency is intrinsically determined, but given that Avicenna’s theory of cognition is entirely deterministic, his theory of will is moderately compatibilist at best, and in strict analysis, it boils down to robust determinism.^13^ Thus, although it is true that the determined truth of ‘Zayd will walk tomorrow’ would render futile Zayd’s deliberation about future perambulation, and for this reason the statement cannot have a determined truth value *now*, it is still the case that every step in Zayd’s deliberation, and therefore his eventual decision, will be determined by occurring motives and considerations, and thus be part of the gapless causal network of the world.

As regards natural events, Avicenna explicitly declares in the *Samāʿ al-ṭabīʿī* that even the rarest events, including those that we deem to take place by chance, are necessitated by their complete causes (Avicenna, *al-Samāʿ al-ṭabīʿī* I.13.8, 85).^14^ In the case of the piece of wood, it is not difficult to see how its future fate is determined by causes extrinsic to it: if it meets with a strong fire, it will inevitably burn (Avicenna, *ʿIbāra* I.10, 74; see also *al-Samāʿ al-ṭabīʿī* I.13.6, 83), and if it is left on the ground, it will just as inevitably decay over time. Finally, Avicenna often mentions voluntary acts and contingent natural events together in explicit commitments to determinism, such as the following:

The volitions that are up to us have come into being after not being, and everything that comes into being after not being has a cause. Hence, every volition that is up to us has a cause. The cause of this volition is not volition regressing to the infinite but extrinsically occurring terrestrial and celestial things. The terrestrial things terminate in the celestial, and the combination of all that necessitates the existence of the volition. As regards chance, it emerges from the collisions of these, and when all matters are analyzed, they are traced back to the principles of their necessitation, which descend from God most high.

(Avicenna, *Ilāhīyāt* X.1.12, 362-363; translation modified.)

Faced with such contradictory pieces of evidence, arguably the most charitable interpretation is one that can explain away one of the seemingly conflicting claims. I maintain that this can be done for the *ʿIbāra* discussion of future contingency. For this purpose, let us recall what Avicenna said about the two ways in which the truth value of statements can be determined: statements are true (or false) (1) intrinsically, or by virtue of essential connections between their constituents, or (2) by virtue of their correspondence to obtaining states of affairs. Future contingent statements cannot be true (or false) on the grounds of (2), because the future states do not obtain yet and thus cannot provide the standard of correspondence by comparison to which we could determine the truth value of the statements. After brief reflection, we realize that their being true on the grounds of (1) is not an option either. The subject terms of future contingent statements are either proper names or concise deictically qualified descriptions that refer to individual things. Proper names do not entail anything, for they are only ways of designating an individual. When it comes to descriptions like ‘this piece of wood’, they do entail certain predicates, namely those entailed by the terms included in the descriptions, but at the same time, we can validly attribute to them many predicates that they do not entail. For instance, ‘this piece of wood is combustible’ is true on its own, because combustibility is concomitant to the essence of wood and thus belongs necessarily to the individual conceived under this description. By contrast, ‘this piece of wood will burn’ is not true on its own, because the question of whether the wood’s capacity to burn will be actualized depends on causal factors extrinsic to the essence of wood. Since these causal factors are not included in the description, the statement does not have a determined truth value on its own.

To put the same point in metaphysical terms, we can say that for Avicenna, modal properties are grounded in essences, or alternatively, in the descriptions of the things to which they are attributed. If an essence or a description entails a property, then that property is necessary to the essence or the thing conceived under that description. If the essence or the description entails the negation of the property, the property is impossible for it. Finally, if the essence or the description entails neither the property nor its negation, the property is merely possible for it, and consequently, external causes are required to determine whether the predication is true in any given case. Contingency of predication is thus always relative to an essence or a description.

In Avicenna’s view, this is where the logician should end her discussion of the truth of future contingent statements. The speculation, to which Aristotle’s remark concerning the sea-battle had given rise, is simply misguided, because it is based on a confused understanding of the division of labour between logical and metaphysical analysis:

The logician, insofar as he is a logician, is not competent to reflect upon the nature of the necessary and the contingent, or to establish contingency. That belongs to another craft. The objective of the logician is only to know the states of truth and falsity, how they are and are not determined, and that in the case of some affairs, [their] determination entails an absurdity that conflicts with what is both evident and widely conceded.

(Avicenna, *ʿIbāra* I.10, 74.)

When a logician begins to speculate about the consequences of the determined truth value of future contingent statements, he puts on the metaphysician’s hat, which is an ill fit for him. The logician should be exclusively concerned with the question of how the truth of statements is determined, and whether future contingent statements can have a determined truth value. The tools of logic provide him with perfectly adequate means of solving this question: by excluding all the alternatives, one can confidently say that future contingent statements do not have determined truth values. By contrast, the tools of logic are inadequate for solving the question of whether reality, considered independently of anything we say about it, is necessarily the way it is.

Thus, statements about the future states of individual things are contingently true because their subjects do not entail their predicates. This kind of modality is entirely dependent on the compass of our description of the subject. Take Avicenna’s own example, ‘this piece of wood will be destroyed by burning’: if we replace the subject with ‘this piece-of-wood-that-Zayd-will-cast-to-the-pyre’, then *ceteris paribus*, the proposition’s modal status will change to necessary, because the predicate will now be entailed by the subject. Another way of putting this point is to say that logic deals with *epistemic* modality, understood as a property of statements taken in isolation – whether, barring information extrinsic to a sentence, the sentence’s truth can be determined intrinsically, or by means of the intensional meanings of its terms. Of course, in some cases, such as the essential truths that are the subject matter of science, epistemic and metaphysical modality coincide, but this does not hold of all sentences, and especially not of sentences concerning future states of affairs that can only be known by immediate acquaintance or by knowing their causes.

Understood as a property of statements, epistemic modality is not affected by a putative cognitive subject’s learning or lack thereof, but only by modifications to the statement’s terms, and thereby to the statement itself. Nevertheless, the epistemic modality of statements concerning the future states of individual things does depend on our descriptions of those individuals, and particularly on whether those descriptions mention the causes relevant to its future states. For this reason, the fact that a state of affairs *under a certain description* is contingent only means that that description lacks the relevant causal information; most importantly, it does not entail the metaphysical contingency of the real state of affairs denoted by that description.^15^ Instead, Avicenna consistently holds throughout the logical discussion, that in themselves, or in proper metaphysical analysis, all factually obtaining states of affairs do so necessarily.

## God’s knowledge of particulars

The fact that the contingency of future contingent statements is exclusively epistemic becomes even clearer once we contrast our way of knowing particulars with that of God. According to Avicenna’s famous phrase, God does know particulars, but only “in a universal way” (*ʿalā naḥwin kullī*, *min jihatin kullīya*) or “as universal” (*min ḥaythu hiya kullīya*).^16^ This is commonly taken to entail that God does not know individuals, because there are no universal concepts of individuals, but only of their species and their concomitant properties (Marmura, “Some Aspects of Avicenna’s Theory”; echoed by Adamson, “On Knowledge of Particulars”). God thus only knows the general ontological blueprint of the world, and although this includes full knowledge of celestial things and events, the sublunary world of generation and corruption, the sphere of future contingency, is excluded from His knowledge.

This interpretation takes its cue from Avicenna’s standard example of an individual event that God knows in a universal way, namely, an individual eclipse. In Marmura’s reconstruction, the celestial bodies are the sole instantiations of their species, and for this reason, God knows them by knowing their species. Furthermore, since the motions of the celestial bodies, and consequently all celestial phenomena, are concomitant to the species of those bodies, God knows them as well. Hence, God is like an omniscient astronomer who can calculate the emergence of each eclipse from His combined knowledge of all celestial motions. However, His calculation only yields atemporal knowledge of the eclipses; in terms of J. Ellis McTaggart’s influential distinction between the A-series and the B-series of time (McTaggart, “The Unreality of Time”), Avicenna holds that God has B-series knowledge of the individual eclipses, because He knows them from outside of time as a series of events that follow one another, whereas a human astronomer, who considers them from within time, knows them as past, present, or future, and consequently has A-series knowledge of them. Unlike the celestial bodies, however, sublunary individuals are numerically distinct instantiations of species that they share, and consequently, they cannot be known simply by knowing their species. Furthermore, each of them has properties that are due to causes extrinsic to their essence, and thus knowledge of their essence does not include knowledge of these properties. Hence, God’s knowledge of particulars ‘in a universal way’ is limited to celestial things and events.

Notwithstanding the prominence of this interpretation, I believe it fails to adequately capture Avicenna’s intention. In the posthumously compiled *addenda* to the *Shifāʾ*, Avicenna repeats the view that God knows particulars in a universal way, but consistently adds the qualification that this sort of knowledge amounts to knowing a thing or an event *by means of its causes*. For a particularly explicit formulation of the idea, consider the following text:

The First understands His own essence and He understands His concomitants, which are intelligible [things] that exist from Him and the existence of which is caused by His understanding them. He understands the concomitants of those existing [things], and among those concomitants there is time and motion. When it comes to corruptible [things], He understands them as corruptible from the point of view of their reasons and causes (*min jihati asbābihā wa-ʿilalihā*), just as you understand a corruptible [thing] when you understand it from the point of view of its causes. For an example of that, if you understand that whenever the matter in the veins is infected, it is followed by fever, and [then come to] know, together with that cause and reason, that there exists an individual for whom this happens, you will judge that that individual is in fever. This judgment will not become false (*lā yafsidu*), even if the subject were to vanish (*fasada*).

For another thing, the intelligible [things] that follow from sensible [things] are not perceived through a cause. All that is sensed is intelligible in one respect, although it is not intelligible with respect to reasons and causes, for it is temporal and changing. The First is to be judged differently from us, for time is intelligible to Him in every respect, whereas it is sensible to us in one respect and intelligible in [another] respect. Individuated [things] are also intelligible in a certain respect, for a certain position is necessitated by a cause, and that cause and that position can be understood as universal. The First only understands these things in the order of their existence, perceiving them all in their order. Even though the individual is individual in existence, that individual is intellectual for Him, insofar as He perceives it from its causes. In our case, too, if we perceive the causes of some individual, we judge that whenever those causes exist, the individual will exist, for the individuality of which those causes are causes. We, however, do not know which causes lead to the existence of these causes, and the preceding causes are infinite. For the First, those causes are intelligible in their order and hierarchy, and not a single existing [thing] escapes His knowledge.^17^

(Avicenna, *Taʿlīqāt* §636, 358-360)

This passage, like its many parallels, explicitly states that individual things and events in the sublunary world of generation and corruption, such as a person’s catching fever, are knowable if one knows their causes, such as the infection in the person’s veins. It also explicitly states that God knows all such causal relations, and thereby all individual things and events, including in the sublunary world. Finally, this is because all causal series ultimately trace back to God.

Such knowledge through causes is perfectly compatible with knowledge ‘in a universal way’. Avicenna expresses causal relations by conditional propositions that have the general form of *if x, then y*. The different logical form notwithstanding, knowing a thing by knowing its cause is knowledge for the same reason as predicative knowledge that captures relations of constitution or entailment between a subject and its predicate: the causal relation known is unchanging, necessary, and obtains universally – given the cause, the effect *necessarily* follows. The universality of causal knowledge is also expressed in our passage, when Avicenna says that knowing the connection between the venous infection and the fever amounts to knowing that “*whenever* (*kullamā*) the matter in the veins is infected, it is followed by fever”.^18^ Thus, knowing individuals through their causes is a case of knowing them in a universal way. What is more, God’s causal knowledge is not merely hypothetical, for by knowing Himself as the first cause of all worldly processes, He knows that the antecedent, and thereby the consequent, of every causal conditional is true. The sum of all this is that Avicenna employed celestial things as his example of knowing a particular in a universal way, not because they are the sole instantiations of their species and thus accidentally knowable by knowing their species, but because they provide a lucid example of what it means to know an individual through its causes.^19^ The complete cause of a celestial phenomenon, such as the lunar eclipse, consists of a relatively small number of factors, which makes it an illustrative example of the more general point.

One might want to object at this point by saying that unlike the celestial phenomena, which are fully necessitated by the uniform motion of the celestial spheres, sublunary causal connections can be, and frequently are, broken down by various circumstantial factors. For instance, people’s immune systems vary in strength, and a particularly strong system may prevent an infection from causing fever, even if the causal connection held in most cases. Thus, the argument goes, individuals are never knowable in the strict sense of the word, because we can never know the complete set of causes and circumstantial conditions that jointly determine an individual event. This is true of human epistemic subjects, because as Avicenna says in the above passage, the causal ancestry that enters the determination of an individual thing or event is infinite, whereas we are finite epistemic subjects. By contrast, however, it need not be true of a perfect epistemic subject, such as God. Avicenna expressly holds that every individual thing and event *is* necessary by virtue of its complete cause, and thus it should be in principle knowable, if one had access to that complete cause.^20^ Since all causes eventually lead back to God, He has access to the complete cause of every individual thing and event, and since there is no limit to His knowledge, an infinite causal ancestry is not a problem.^21^

Now, the difference between our way of knowing individual things and events and God’s way of knowing them does not merely concern the completeness of our knowledge of their causes. When we know an individual by knowing its causes, like a physician does when making a particular diagnosis, we know the individual by having sense perception of it, and our causal knowledge only amounts to subsuming the individual under a certain universal description. Our knowing the individual through sense perception entails knowing it from a certain temporal perspective, whereas God’s knowledge through causes is atemporal. Avicenna explicates this when speaking about the example of eclipse:

Hence, the Necessary Existent’s knowledge cannot be of the particular insofar as it can be pointed at (*mushāran ilayhi*), like the eclipse when we say, “this eclipse at which one points”, or “the eclipse that takes place today or tomorrow”, for He is also acquainted with the tomorrow in a universal way. He is acquainted with it as [being] after such and such time or such and such motion, so that He is not acquainted with it as being pointed at.

(Avicenna, *Taʿlīqāt* §1, 6.)

Again, God’s atemporal knowledge of temporal individuals is in terms of unchanging B-series relations of temporal priority and posteriority. By contrast, we know the same things from within time, or in terms of the A-series, as something that occurred in the past, presently occurs, or will occur in the future.^22^

Considered in tandem, these two differences regarding the completeness of the known causes and the relation to time have important consequences for our discussion of future contingency. As we saw, future contingent statements are always statements about individual things. From a logical point of view, this means that such statements refer to their individual subjects deictically: *this* thing *here*, and especially *now*. The requirement of deictic reference becomes clear when the set of concepts used to describe the subject is not sufficient on its own to single out an individual. For instance, ‘*this* piece of wood’ refers to an individual thing, and consequently can function as a subject term in a future contingent proposition, whereas ‘piece of wood’ does not, and only allows us to form an absolute proposition, which does not yield a pair of future contingent contradictories. Most importantly, the reference to time is deictic in the same sense, and since deictic reference to individuals presupposes a spatiotemporal location, it is excluded from God. In other words, the futurity of future contingents is only due to our epistemic perspective.^23^

The same holds of the contingency of future contingents. If one’s access to the individual is through the entire causal network that necessitates it, then the causes that necessitate the individual must be part of its description.^24^ To put this another way, God knows the individual thing or event under its complete description, which includes all the features that constitute it *as an individual*.^25^ Now, it should be evident that a complete description of an individual will have very different logical properties from those of our limited descriptions. Any predicate, including predicates we would attribute in future tense, that is truthfully predicable of the individual conceived under its complete description will be entailed by the description, and thus necessarily true of it. To use Avicenna’s own terms, statements that include complete descriptions as their subject terms will have determinate truth values ‘on their own’, independent of extrinsic states of affairs.

This means that for an epistemic subject who knows individuals by virtue of their complete causes, there is no such thing as future contingency. The comparison to God’s way of knowing individuals thus shows that the future contingency discussed in logic, that is, the indeterminacy of the truth value of statements about individual things and events in the future, derives not from the things known but from our way of knowing them. In other words, the contingency of future contingents is exclusively epistemic, and the reason why we must not draw metaphysical conclusions from this logical observation is that it has none.

## Conclusion

If the foregoing is on the right track, future contingents are not a problem for a necessitarian interpretation of Avicenna. At best, they provide an opportunity to highlight the level of sophistication of Avicenna’s modal metaphysics. The distinction between epistemic and metaphysical modality shows that he took seriously the intuitions that seem to support indeterminism, but it also shows that he tackled them by drawing a strict boundary between the limits of human knowledge and the determination of reality.

On the other hand, the contrast of human knowledge with God’s knowledge allows us to draw further support for necessitarianism. If God knows all particulars through their causes, and if He knows these causes by knowing Himself as their ultimate causal principle, then there is little room for alternative states of affairs. Given that there is only one God and that from what is absolutely one in all respects, only one effect can proceed, there can be only one actual world.^26^ Furthermore, since God creates the world by intrinsic necessity, He does not consider counterfactual alternatives. Thus, for Avicenna, there is only one world that is thoroughly determined by God’s atemporal knowledge of it, and no room for counterfactuals, if not in human imagination.

All this notwithstanding, Avicenna remains a realist about contingency, which has understandably led some of his readers to reject the claim of his necessitarianism. These two stances, however, need not be contradictory. This is because Avicenna’s modal metaphysics is grounded in his essentialism; for him, something is contingent in the sense that its essence does not necessitate its existence, or to put this another way, its essence does not exist necessarily. By the same token, a property contingently belongs to a thing if the thing’s essence neither necessitates nor excludes that the property belongs to the thing. It is important to note, however, that an essence and its factually existing individual instantiation have radically different modal properties: where the essence exists contingently and possesses many properties contingently, the individual constituted not only by the essence but also by the properties extrinsic to the essence, exists necessarily and necessarily possesses all the properties it does. Of course, the necessity of the individual’s existence is not due to its essence but to extrinsic causes, and ultimately to God, but it is necessity none the less. Nothing in a factually existing individual is contingent. This distinction between the different modal properties of the essence and its individual instantiation is a consequence of Avicenna’s distinction between essence and existence. I am not saying anything particularly new here, but it seems to me that this entailment is sometimes insufficiently understood in the debate about Avicenna’s necessitarianism.

## Footnotes

1 For the PSR in Avicenna, see Richardson, “Avicenna and the Principle of Sufficient Reason”, who remains hesitant about Avicenna’s commitment to the principle’s necessitarian consequences. For more straightforward assertions of Avicenna’s necessitarianism, see Goichon, *La distinction*, 162-163; Marmura, “The Metaphysics of Efficient Causality”, 172-187; Michot, *La déstinée*, 61-64; Frank, *Creation and the Cosmic System*, 23-24; Belo, *Chance and Determinism*, 91–120 and 225-232; and De Cillis, *Free Will and Predestination*, 36-40.

2 For a brief critical discussion of these arguments, see Kaukua, “Freedom and Responsibility in Avicenna”.

3 I have argued against the voluntarist interpretation of Avicenna in Kaukua, “Freedom and Responsibility in Avicenna”.

4 For similar assessments of Avicenna’s modal logic, see Marmura, “Divine Omniscience”, 91-92; and Thom, “Logic and Metaphysics”. Notice that Bäck concludes in somewhat hesitant terms by suggesting that due to Avicenna’s commitment to a necessary ground for all existing things (God), logical and causal determinism eventually seem to coincide, which entails necessitarianism. As I will argue towards the end of this paper, I think this is correct, but I do not believe it entails any inconsistency in Avicenna’s modal metaphysics.

5 In this context, *bi-ʿaynihi* thus comes very close to Fārābī’s phrase *ʿalā l-taḥṣīl*, on which see Adamson, “The Arabic Sea-Battle”, 172-180. I am grateful to Yusuf Daşdemir and Francesco Zamboni for their helpful comments on Avicenna’s terminology here.

6 Thus, we can dispense with the principles concerning quantification as well as the complexities related to indefinite propositions (such as ‘man is an animal’).

7 Avicenna, *ʿIbāra* I.10, 67: “When it comes to the essence of an individual subject, complete difference in terms of affirmation and negation suffices, *because the subject is determined* (*li-taʿayyuni l-mawḍūʿ*)” (my emphasis).

8 It is important to note here that in many quantified statements, the two ways of truth determination coincide, for in a robustly essentialist metaphysics like that of Avicenna, essential truths of the first kind are also factual states of affairs constituted by the real referents of our essence concepts. (I thank my reviewer for alerting me to this point.) I still claim, however, that Avicenna here distinguishes between two ways of truth determination, for in the case of statements about individuals, only one of them holds.

9 An exception must be made for descriptions that are used to denote individuals, such as ‘this piece of wood’, for terms like ‘wood’ have similar entailments regardless of whether they are used to designate an individual or in quantified propositions. I will return to this in what follows.

10 In addition to all this, Avicenna’s reference to necessary things at the end of the quote strongly suggests that he remains committed to the PSR: all things that exist, exist necessarily, and this either by virtue of their essence or by virtue of an extrinsic cause.

11 One might think that statements like this constitute an exception to Avicenna’s condition that to form contradictory pairs, future contingent statements must refer to a specific future time. It is true that no such reference is required for genuine contradiction to hold between ‘this piece of wood will be destroyed by burning’ and ‘this piece of wood will not be destroyed by burning’. On the other hand, expressions like ‘will be destroyed’ make an implicit reference to a vaguely specified time, because the destruction of the piece of wood is not a repeatable event. Restrictions of space prevent me from further discussing this complication.

12 This is perceived as an inconsistency by Marmura, “Divine Omniscience”, 91-92; and Bäck, “Avicenna’s Conception of the Modalities”, 246-249.

13 For an extended argument in this vein, including references and critical discussion of alternative interpretations, see Kaukua, “Freedom and Responsibility in Avicenna”.

14 For an extended discussion, see Belo, *Chance and Determinism*, 21-53.

15 This is also why Avicenna is suspicious about drawing metaphysical conclusions from mere conceivability. On this topic, see McGinnis, “A Penetrating Question”; and Kukkonen, “Ibn Sīnā and the Early History”.

16 Avicenna, *Ilāhīyāt* VIII.6.15-20, 288-289. For a detailed discussion of the reasons why God’s knowledge must be universal, see Zadyousefi, “Does God Know the Occurrence”, and “Does God Know That the Flower”.

17 See also Avicenna, *Taʿlīqāt* §1, 5-10; §14, 34-36; §§16-18, 36-39; §20, 39-40; §30, 48-49; §§32-36, 51-54; §§245-246, 166-169; §339, 215-216; §§595-596, 334-335; §598, 335-336; §600, 337; §612, 342-343; §616, 345; §620, 347; §§622-623, 348-349; §629, 353; §§633-634, 355-357; §639, 361; §653-655, 367-369; §835, 453-454; §§861-862, 467-469.

18 For the universal quantification of implicative statements, see Avicenna, *Qiyās* V.4, 263-265.

19 This has been argued before me by Zghal, “La connaissance des singuliers”.

20 Cf. Avicenna, *Dānish-nāma: Ilāhīyāt* 31, 88-90. Adamson, “On the Knowledge of Particulars”, 275-276, recognizes this possibility but rejects it because it would lead to determinism. His ground for rejecting determinism in Avicenna is Ivry, “Destiny Revisited”, who argues that prime matter is a source of indeterminacy in the Avicennian universe. I do not believe that this argument stands scrutiny, for the actual existence of matter is always determined by elemental forms, the emergence of which is causally determined by the celestial motions. For a more detailed discussion, see Kaukua, “Freedom and Responsibility in Avicenna”.

21 One might think that an infinitely complex series of known causes is an ordered actual infinite that an Aristotelian must reject. This is a genuine problem, and I think Avicenna tried to solve it by means of his famous distinction between knowing an answer one has never explicated and discursively explicating that answer (see, for instance, *Taʿlīqāt* §1010, 583-584, and cf. §30, 48-49; §32, 51-52; §612, 342-343; §620, 347; §§633-634, 355-357; §639, 361; §§653-655, 367-369; and §847, 459-460). On the other hand, the problem is not specific to my interpretation, for it also holds of the knowledge of celestial phenomena, which can be numerically distinct only by virtue of the infinite prior phases of celestial motion.

22 Nusseibeh, “Avicenna: Providence”, conceives of this difference in terms of the Russellian knowledge by acquaintance and knowledge by description.

23 One might insist that God does not know individuals in the proper sense of the word, because He lacks A-series access to them as things that exist *here* and *now*. I grant this, but I would also like to point out (1) that arguably this is precisely why Avicenna says that God knows the things that we take to be individuals *in a universal way*, and (2) that no addition or subtraction of information hinges on the difference of temporal perspective – there is nothing God would learn by knowing things in terms of the A-series.

24 On the transformation of causal connections into definitional formulas (which can function as subject terms in statements comparable to future contingent statements), see Avicenna, *Burhān* IV.4, 289-290; and for discussion, Strobino, *Avicenna’s Theory of Science*, 298-302.

25 Incidentally, this coincides with the way in which most post-Avicennian *mutakallimūn* interpreted Avicenna’s theory (see Benevich, “God’s Knowledge”, 13-26).

26 For this principle in Avicenna, see Amin, “From the One, Only One Proceeds”; and Dadikhuda, ‘Rule of the One’.
